# A Mini-Review on the Common Antiviral Drug Targets of Coronavirus

**DOI:** 10.3390/microorganisms12030600

**Published:** 2024-03-17

**Authors:** Jun Wang, Qinghe Zhu, Xiaoxu Xing, Dongbo Sun

**Affiliations:** College of Animal Science and Veterinary Medicine, Heilongjiang Bayi Agricultural University, No. 5 Xinfeng Road, Daqing 163319, China; wj057596@163.com (J.W.); qinghezhumy@126.com (Q.Z.); xingxiaoxu5451@163.com (X.X.)

**Keywords:** coronavirus, virus targets, common mechanism, common antivirals, broad-spectrum

## Abstract

Coronaviruses in general are a zoonotic pathogen with significant cross-species transmission. They are widely distributed in nature and have recently become a major threat to global public health. Vaccines are the preferred strategy for the prevention of coronaviruses. However, the rapid rate of virus mutation, large number of prevalent strains, and lag in vaccine development contribute to the continuing frequent occurrence of coronavirus diseases. There is an urgent need for new antiviral strategies to address coronavirus infections effectively. Antiviral drugs are important in the prevention and control of viral diseases. Members of the genus coronavirus are highly similar in life-cycle processes such as viral invasion and replication. These, together with the high degree of similarity in the protein sequences and structures of viruses in the same genus, provide common targets for antiviral drug screening of coronaviruses and have led to important advances in recent years. In this review, we summarize the pathogenic mechanisms of coronavirus, common drugs targeting coronavirus entry into host cells, and common drug targets against coronaviruses based on biosynthesis and on viral assembly and release. We also describe the common targets of antiviral drugs against coronaviruses and the progress of antiviral drug research. Our aim is to provide a theoretical basis for the development of antiviral drugs and to accelerate the development and utilization of commonly used antiviral drugs in China.

## 1. Introduction

Antiviral drugs are widely recognized as a valuable approach for preventing and controlling viral diseases, particularly in the acute treatment of new outbreaks. These drugs can significantly alleviate the disease, reduce mortality, and serve as an effective alternative or supplement to vaccines. Currently, there are several main types of antiviral drugs available, including inhibitors of viral adhesion, internalization, and release; drugs that restrict polymerase or protease activity; and nucleoside/nucleotide reverse transcriptase inhibitors and integrase inhibitors [1]. Most of these antiviral drugs target a particular protein of a particular virus, which offers the advantage of high specificity and minimal harm to the host. However, the downside is that the mutable nature of viruses can easily lead to drug resistance, owing to mutations in the viral drug target, which also means that existing drugs are of limited help in controlling viral infections. In addition, although some antiviral drugs effectively suppress viral infections, they often come with drawbacks such as high toxicity, low potency, adverse drug reactions, and off-target side effects [2]. Development of virus-specific drugs first requires addressing the basic biology of the virus to find a suitable target, followed by medicinal chemistry studies, compound screening, optimization, animal studies, and ultimately clinical trials. This process can be lengthy, cumbersome, and difficult. Most of the drugs that have been developed reduce clinical symptoms and symptom duration but cannot completely remove the virus. The drugs must be administered regularly, which greatly limits their clinical application [3]. This, coupled with the limited number of disease proteins suitable for drug development, has resulted in the slow progress of research on antiviral drugs. There are still no effective antiviral drugs available for many viral infections, which has been a common and critical problem in this field.

The discovery of antiviral drug targets has been advanced with the revelation of viral life-cycle mechanisms, such as viral invasion, genome transcription, replication, assembly, and release; mechanistic revelations of virus–host interaction mechanisms; and the rapid development of antiviral drug screening and development technologies. Currently, anti-coronaviral drug targets mainly focus on three aspects: viral invasion, replication, and release [4]. In terms of viral invasion, the design of antiviral drugs mainly revolves around interfering with the fusion of the viral vesicle membrane with the host cell membrane or inhibiting host receptor proteins. For example, antiviral drugs inhibit the coupling of viruses to relevant host receptors by binding to specific viral proteins, inhibiting viral entry into target cells in the form of receptor binding [5,6], or inhibiting viral replication by specifically inhibiting the contact, adhesion, and fusion of viral lipid vesicle membranes to host cell membranes [7,8]. In the context of viral replication, the design of antiviral drugs is centered around interfering with the function of viral replication-associated proteases. For example, some antiviral drugs can inhibit viral replication by targeting viral replication-related enzymes. In terms of viral assembly and release, antiviral drugs inhibit virus formation by inhibiting the modification of cytosolic proteins by viral proteases. These targeting efforts have revealed small-molecule compounds, chemically synthesized drugs, natural products, peptides, and other antiviral drugs, which have enhanced antiviral research and opened the door to the development of antiviral drugs with broad-spectrum activity.

In addition to vaccines, research on antiviral drugs is one of the key strategies for coronavirus prevention and control. Although different viruses replicate in different ways, there are several common stages in the viral replication cycle. These include viral adherence, invasion, exsiccation, biosynthesis, assembly, and release [9,10]. Based on key replication mechanisms, such as the viral life cycle, a series of antiviral drug studies have been performed by blocking any one, or a combination of, the aforementioned viral life-cycle stages, resulting in effective drugs. However, coronaviruses have proven to be challenging. Their positive-strand RNA can be used both directly as a translation template and as a template for the synthesis of negative-stranded RNA, which in turn replicates the daughter positive-stranded RNA, thereby generating a large number of copies of the viral genome that are used to generate progeny viruses that infect new host cells. The replication of one virion can lead to 100 to 1000 progeny. In addition, coronaviruses have one of the largest known genomes among RNA viruses. This large genome instills a high mutation frequency during replication [11]. This aspect, coupled with the increased risk of cross-species transmission of coronaviruses due to increased human–animal interactions, has constrained the rapid development of coronavirus antiviral drugs. In seeking to overcome this constraint, the utilization of common antiviral targets, as well as the development of multi-combination antiviral drugs based on common targets, which has been promising for other viruses, is a potentially valuable direction for coronaviruses. Identification and exploitation of common coronavirus drug targets is central to this research.

In this review, we focus on the life cycle of coronavirus-infected hosts and combine the research results of scholars from various countries to consider the common antiviral drug targets of coronaviruses. The aim is to provide reference data for the clinical treatment of coronavirus infections and the timely development of broad-spectrum antiviral drugs.

## 2. Pathogenic Mechanisms of Coronavirus

Coronaviruses belong to the order *Nidovirales*, the family *Coronaviridae*, and the genus *Coronavirus*. They are a diverse group of enveloped viruses with a linear, positive-sense, single-stranded RNA genome [12]. The genus *Coronaviridae* is further divided into the family *Coronaviridae* and the subfamily *Coronavirinae.* This subfamily is in turn divided into four variants: α-, β-, γ-, and δ-coronaviruses [13]. In recent years, large-scale outbreaks of human and animal coronavirus infections have occurred, which have threatened the health and safety of humans and animals [14,15,16].

Vaccination is the preferred strategy for preventing coronaviruses. However, given the broad distribution range, numerous prevalent strains, and genetic diversity of coronaviruses, and increased interaction between humans and animals, the risk of cross-species transmission of coronaviruses has increased [17]. The development of new vaccines always lags behind virus mutations, which may reduce the effectiveness of these vaccines and challenge efforts to prevent coronavirus infections.

**Figure 1:**
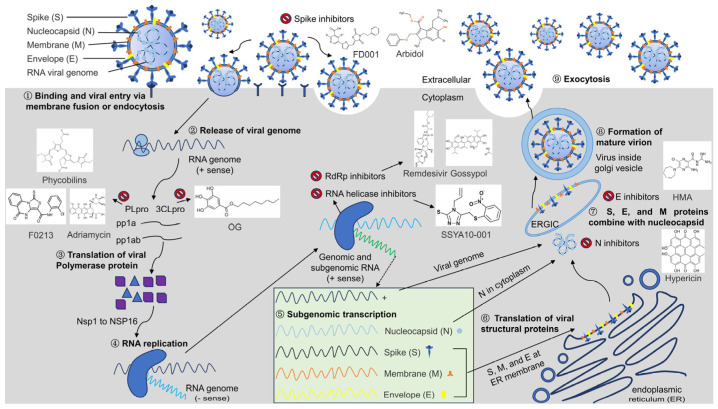
Life cycle of coronavirus and drug targets. The sequence of events, from host cell recognition through the release of the new virion, is represented graphically as steps 1 to 9. The structure of the drug was obtained from PubChem (pubchem.ncbi.nlm.nih.gov).

Antiviral agents are the last line of defense against viral diseases. Screening for safe, inexpensive, and common antivirals remains crucial for current existing and emerging coronavirus diseases. Generally, congeneric viruses share similar replication strategies and proteins, which are common targets for the development of antivirals. As seen in Figure 1, the key proteins involved in the transmembrane entry, transcription, translation, assembly, and release of new coronavirus particles may be targets for anti-coronavirus drug development [18]. Invasion is the primary step of viral infection and includes receptor recognition, endocytosis, and membrane fusion [19]. Initially, the virus attaches to the host cell, and subsequently, the glycoproteins on the envelope of the virus attach to the receptor/co-receptor molecules on the host cell membrane and enter the host cell through endocytosis by the host cell and membrane fusion. Next, the capsid of the internalized virus is degraded by the host cell enzymes, and its genetic material is released into the host cytosol. Following viral entry, the viral genome is cleaved by papain-like proteases (PLpro) and 3C-like proteases (3CLpro) to produce nonstructural proteins that are involved in viral transcription and replication [20]. The positive-strand RNA of the coronavirus is then translated to produce the negative-strand RNA polymerase precursor protein, followed by the production of RNA-dependent RNA polymerases through a process of protein hydrolysis, and further, the generation of full-length antisense negative-stranded templates for the synthesis of subgnomic mRNA from negative-stranded subgenomic templates, through the action of RNA polymerases. Utilizing host-cell protein-synthesis mechanisms, translation of subgenomic mRNAs produces viral structural proteins, which are subsequently transported out in the endoplasmic reticulum (ER), undergoing assembly of viral membrane proteins and the formation of vesicles. Next, in the endoplasmic reticulum–Golgi intermediate, the nucleocapsid-coated positive-stranded RNA is recognized by the membrane proteins and the vesicles invaginated, encapsidating the RNA to form a new virion, which is finally released into the extracellular space by outgrowth or cytotoxicity, and the released virus then goes on to infect other cells for the next round of replication [21,22,23]. Accordingly, small-molecule inhibitors or peptides that target key proteins in the common links of coronavirus replication have become common antivirals to thwart coronavirus infection (Table 1). In this review, we summarize the progress of antivirals against common targets to provide a reference for the clinical therapy of coronavirus infection and the development of common antivirals.

## 3. Common Drugs Targeting Coronavirus Entry into Host Cells

**Figure 2:**
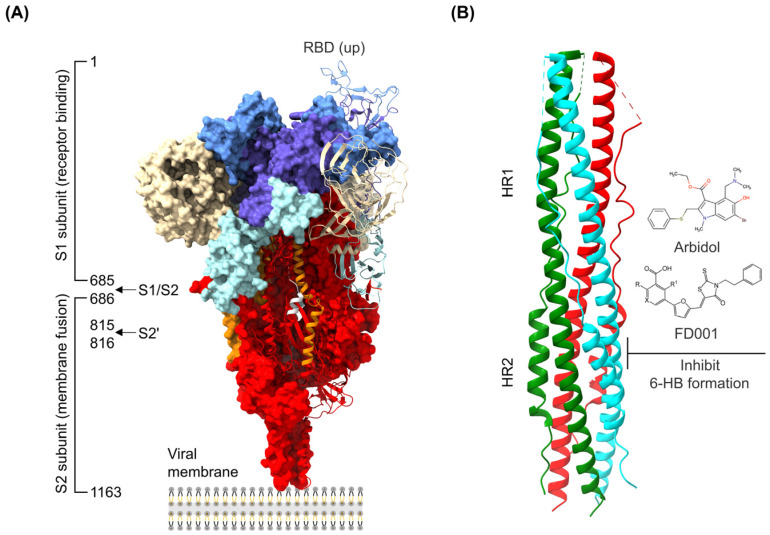
Common antivirals targeting the coronavirus spike. (**A**) Side view of the pre-fusion structure of the SARS-CoV-2 spike protein (PDB ID 7WEA) with a single receptor-binding domain in the “up” state. (**B**) Three pairs of HR1/HR2 of the trimeric S protein (colored green, cyan, and red) form a six-helix bundled coiled-coil structure in the post-fusion state after cleavage at the S1/S2 boundary (PDB ID 6LXT). Arbidol and FD001 inhibit formation of the six-helix bundle (6-HB).

Coronavirus infection depends on the S protein, a type I transmembrane glycoprotein that binds host cell receptors and induces virus–cell membrane fusion, which plays a crucial role in virus invasion [39]. The coronavirus S protein contains two subunits, S1 and S2. The S2 subunit is anchored to the viral membrane through a transmembrane region. This subunit contains the essential components required for the membrane fusion process, enabling the virus to bind to the cellular membranes and undergo fusion (Figure 2A) [40,41].

Drugs that target coronavirus entry into host cells are mainly small-molecule compounds and peptides. For example, the small-molecule compound FD001 and its derivatives [24] and Arbidol [7] have broad−spectrum antiviral activity against various coronaviruses by inhibiting the formation of heptad repeats of the S2 subunit, thereby disrupting viral−mediated membrane fusion (Figure 2B). Common antiviral drugs designed based on the mechanism of targeting viral invasion have the advantage of being able to directly block the binding of viruses to the host. This in turn blocks viral entry into the host cell from the source, abrogating viral replication and proliferation. Thus, the chance of viral infection is markedly lessened. However, coronavirus S protein has the highest mutation rate of any coronavirus protein. The mutation rate of its receptor-binding region exceeds 85%. The S protein is poorly conserved among viruses and has a complex structure. These aspects can often lead to problems such as the emergence of drug resistance. Furthermore, different coronaviruses recognize different receptors. Thus, small-molecule drugs targeting S protein may be ineffective against certain viruses. Future studies could use the relatively conserved region of the S protein to design drug combinations against common targets of different S variant genotypes or against antiviral drug targets of different genotypes of the S protein. In addition, further optimization of the molecular structure of antiviral drugs could facilitate the development of antiviral drugs based on the coronavirus S protein that feature better pharmacodynamic properties.

## 4. Common Drug Targets against Coronaviruses Based on Biosynthesis

Upon entry of a coronavirion into the host cell, the viral genome is released into the cytoplasm or nucleus, and the replicase polyproteins pp1a and pp1ab are processed into 16 nonstructural proteins by PLpro and 3CLpro, respectively. In this process, 3CLpro, PLpro, RNA-dependent RNA polymerase (RdRp), and RNA helicases are all essential for both viral replication and host cell control. These proteins are thus attractive targets for the development of antivirals. Furthermore, targeting the biosynthetic pathway may be an effective way of discovering common antivirals. The advantage of antiviral drugs targeting these proteases is that, although the virus has entered the cells, the viral polyprotein cannot be cleaved when the antiviral drug binds to the corresponding target, resulting in the inability to form a new generation of viruses, thus inhibiting viral replication and greatly reducing the viral load. However, the disadvantage is that certain proteases, such as PLpro and coronavirus deconjugating enzymes, have structural similarities with the host enzymes, and antiviral drugs are also able to recognize and inhibit some of these enzymes in the host. Thus, there are certain off-target and side effects and a possibility that the risk of a harmful response to viral infections (cytokine storms, etc.) will increase after the off-target event, necessitating caution in the choice of drugs. In addition, as discussed with the S protein, the emergence of new mutants creates a potential risk of drug resistance, and the structure of the compound needs to be further optimized to address off-target side effects in future research and development.

### 4.1. Coronavirus PLpro as a Common Target for Antivirals

Coronavirus PLpro is a domain of nonstructural protein 3 (Nsp3) that cleaves pp1a and pp1ab and mediates the deubiquitination of antiviral proteins and deISG15ylating (interferon-induced gene 15) activity. Thus, PLpro participates in the regulation of the host antiviral innate immunity [42]. The structure and function of PLpro are highly conserved in coronaviruses: severe acute respiratory syndrome-coronavirus-2 (SARS-CoV-2) has 83% sequence homology with SARS-CoV, 30.3% with Middle East respiratory syndrome (MERS)-CoV, and 20.77% with severe acute diarrhea syndrome (SADS)-CoV [43,44]. Given the dual functionality of PLpro, coronavirus PLpro is an attractive target for the development of common antivirals.

**Figure 3:**
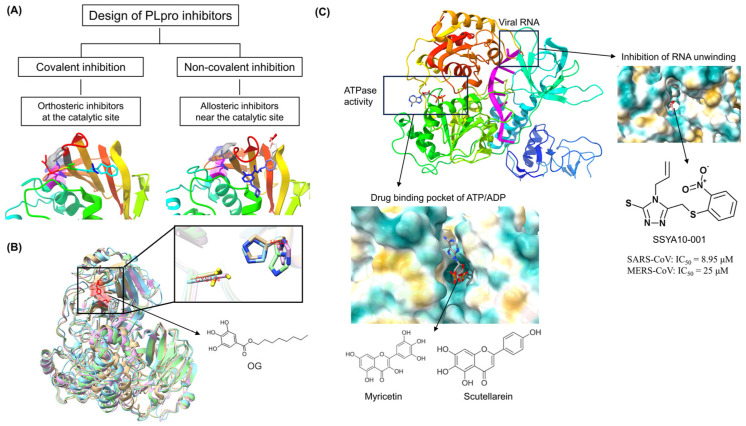
Common antivirals targeting biosynthesis. (**A**) Types of inhibitors that target PLpro. (**B**) Superimposed plots of simulations of PEDV 3CLpro (PDB ID 4XFQ, plum), TGEV 3CLpro (PDB ID 1LVO, tan), PDCoV 3CLpro (PDB ID 7KYU, light sky blue), and SADS-CoV 3CLpro (PDB ID 6w81, pale green). The same catalytic sites (His41 and Cys144) are shown in red. (**C**) Functional domains of RNA helicase (PDB ID 7RDY). One drug−binding pocket is located within the ATP/ADP−binding site where myricetin and scutellarein block the ATP/ADP−binding site. The other is located within the RNA−binding site, where the inhibitor SSYA10−001 blocks the entry of viral ssRNA.

Two main types of covalent drugs target PLpro: non-covalent and covalent inhibitors. Non-covalent inhibitors block the channel used by the substrate to access the catalytic site, thereby inhibiting substrate cleavage of coronaviruses, such as XR8-24 [45], phycobilins [26], and adriamycin [27]. Covalent inhibitors mimic the sequence of the cleavage site to inhibit PLpro activity; examples are VIR250 [45] and F0213 [28] (Figure 3A). Yuan et al. [28] screened a compound library containing 50,080 compounds with diverse structures and identified a non-covalent lead inhibitor, F0213, with broad-spectrum anti-coronavirus activity. F0213 effectively inhibits multiple coronaviruses in vitro, including SARS-CoV, SARS-CoV-2, and their MERS-CoV and hCoV-229E variants. Covalent inhibitors can interact with specific target proteins and form covalent bonds, leading to a change in protein conformation that inhibits the activity of the protein. In contrast, non-covalent inhibitors rely on hydrophobic interactions, hydrogen bonding, and other interactions to bind reversibly to the target proteins. The strength of these interactions is weaker than that of the covalent chemical bonding. Thus, covalent inhibitors tend to be more potent than non-covalent inhibitors. On the other hand, although covalent drugs have a great advantage in terms of their activity, and their irreversible binding can effectively inhibit the reactivation of protein targets, the formation of covalent chemical bonds is not protein-specific. Once an off-target effect occurs, it will result in increasingly toxic side effects. In comparison, the clinical selectivity advantage of non-covalent compounds is greater than that of covalent compounds. Therefore, the future development and optimization of inhibitors should focus on non-covalent inhibitors and consider covalent compounds. The rational optimization of drug structure should consider the balance between reactivity, selectivity, and efficacy to produce safe and effective drugs and overcome the problem of traditional non-druggable targets.

### 4.2. Coronavirus 3CLpro as a Common Target for Antivirals

Coronavirus 3CLpro, also known as coronavirus Nsp5, belongs to the protease associated class of enzymes. 3CLpro cleaves pp1a and pp1ab at 11 distinct cleavage sites. 3CLpro is highly conserved, with 61.5% amino acid homology between the 3CLpro of porcine epidemic diarrhea virus (PEDV), transmissible gastroenteritis virus (TGEV), porcine deltacoronavirus (PDCoV), and SADS-CoV. Protein structure analysis has revealed the marked similarity of the structures of 3CLpro of four swine enteropathogenic coronaviruses [46]. The active catalytic center of PEDV, TGEV, PDCoV, and SADS-CoV 3CLpro is His41-Cys144 (Figure 3B). Furthermore, there is no host homolog of 3CLpro and so the inhibition of 3CLpro does not result in adverse reactions and toxic effects. Therefore, 3CLpro is the most promising target for the development of antiviral drugs.

In analyses involving molecular docking and other computational programs, 3CLpro has been identified as an important target for large-scale screening of common antivirals [29]. For example, a variety of small molecule compounds that include xanthohumol [29], hypericin [30], paxlovid [33], and tomatidine [31] specifically inhibit multiple coronavirus 3CLpro activities by targeting 3CLpro. For example, Paxlovid, an antiviral drug consisting of nimatrevir and ritonavir, which is able to block the replication of SARS-CoV-2 and other coronaviruses by inhibiting 3Clpro, is being evaluated in phase 3 clinical trials and has the potential to be a breakthrough antiviral. To screen for anti-coronavirus drugs and evaluate their effects, we established molecular docking technology and evaluation platforms [38]. Our virtual screening of natural drug-active small molecules using PEDV 3CLpro as a target revealed that octyl gallate (OG) potentially binds to PEDV 3CLpro (CE = 31.147). Furthermore, bio-layer interferometry and fluorescence resonance energy transfer analyses revealed the high-affinity binding of OG binds to PEDV 3CLpro (KD = 549 nM) and the resulting inhibition of PEDV 3CLpro activity (IC_50_ = 22.15 μM). Results also demonstrated that OG significantly inhibited the replication of PEDV, TGEV, PDCoV, and SADS-CoV in vitro and decreased the viral titers of PEDV CV777 and HM2017 strains by 0.58 and 0.71 log_10_ TCID_50_/mL, respectively. In vivo, the cure rate of OG on PEDV-infected piglets reached 75%, with significant reductions in clinical symptoms, pathological damage, and viral loads in the jejunum and ileum of infected piglets. The collective findings indicated the potential of OG as a common antiviral drug for the prevention and control of swine enteric coronavirus [32]. The evidence to date supports the view that novel broad-spectrum antivirals can be created based on the conserved 3CLpro of coronavirus.

### 4.3. Coronavirus RdRp Protein as a Common Target for Antivirals

Coronavirus RdRp, which is encoded by Nsp12, is a key holoenzyme involved in coronavirus replication and transcription [47]. Viral RNA is synthesized within Nsp12, and RNA synthesis can only begin when Nsp7, Nsp8, and Nsp12 form a complex at a ratio of 1:1:2 [48]. The structure and function of RdRp are highly conserved in coronaviruses. SARS-CoV-2 has 98.13% sequence homology with SARS-CoV and 75.51% with MERS-CoV, and the differences in structure are all present outside the active center [49]. RdRp has no homologous enzyme in the host organism. Thus, small-molecule drugs targeting viral RdRp do not interfere with the normal physiological responses of the host cells. In contrast, due to the lack of RdRp counterparts in mammalian cells, its inhibitors have higher potency and fewer off-target and other side effects, and broad-spectrum antiviral drugs that act on the target can concurrently block the majority of viral replication that involves polymerase activity [50]. However, an obvious disadvantage is the presence of a 3′-5′ exonuclease in coronaviruses. The enzyme has corrective functions that ensure a low error rate and high replication fidelity for the viral genome. For nucleoside analogs to bind effectively to the viral genome, they need to evade the correction mechanism of Nsp14. This is another challenge to the development of antiviral drugs.

Various potentially valuable small-molecule compounds have also been discovered based on molecular docking virtual screening and artificial intelligence drug screening [51]. Two examples are remdesivir and gossypol (GOS). Remdesivir is an RdRp inhibitor that has been shown to have broad-spectrum antiviral activity against several virus families, including coronaviruses (MERS-CoV, SARS-CoV, PEDV) and filoviruses [34,52,53,54]. Remdesivir, after entering cells in prodrug form, is converted to the triphosphate metabolite in three steps [55,56], which competes with natural ATP for binding to the RdRp of viruses and then is inserted into the RNA synthesis strand to act as a substrate for RdRp [57]. It competitively inhibits the functional activity of the viral RdRp enzyme and integrates it into the nascent viral RNA, thereby terminating the transcriptional synthesis of the viral genome [58,59]. However, remdesivir requires intravenous administration, probably because of its poor oral bioavailability and short half-life. Future studies will need to optimize the structure to improve oral bioavailability and make treatment more practical. Molnupiravir is the world’s first marketed drug developed by Merck for the treatment of new coronavirus infections. Its mechanism of action is similar to that of Ridecivir, which is to participate in the process of RNA transcription of the virus in the cell and to form the completed transcription of viral RNA with mutations, failing to express the normal proteins and affecting the viral replication cycle, so as to keep the viral load in the body at a low level and realize the effect of the antiviral [60]. GOS reportedly has broad-spectrum anti-coronavirus activity against α coronavirus (PEDV, IC_50_ = 0.99 μM and SADS-CoV, IC_50_ = 2.55 μM), γ coronavirus (IBV, IC_50_ = 1.02 μM), and δ coronavirus (PDCoV, IC_50_ = 1.06 μM) [35]. The potential of GOS as a lead compound for coronavirus treatment warrants further studies.

### 4.4. Coronavirus RNA Helicase as a Common Target for Antivirals

Coronavirus RNA helicase (Nsp13), a member of the SF1 helicases superfamily, has several enzymatic activities, including NTP/dNTP hydrolase, RNA/DNA helicase, and 5′-triphosphatase activity [61]. RNA helicase is one of the conserved nonstructural proteins in coronaviruses, as well as one of the core components of the viral transcription/replication complex. The enzyme is involved in RNA processing and quality control to maintain the replication of the RNA genomes of coronaviruses [62]. Therefore, RNA helicase is one of the most important targets for antiviral design. 

FRET has been used to screen several small-molecule compounds, such as SSYA10-001, myricetin, and scutellarin, for their ability to inhibit helicase RNA unwinding or ATPase activity (Figure 3C) [63,64]. SSYA10-001 blocks coronavirus double-strand RNA (dsRNA) and dsDNA unwinding activities by influencing conformational changes or nucleic acid translocations during the reaction. These activities inhibit the replication of SARS-CoV (IC_50_ = 8.95 μM), MERS-CoV (IC_50_ = 25 μM), and MHV (IC_50_ = 12 μM) [36]. SSYA10-001 is a potential candidate for further development as a common antiviral drug. However, coronavirus helicases share similar substrate-binding structures and functions with some host helicases, such as DDX helicases [65], which may increase the risk of off-target effects. Further compound structure optimization is needed to address these off-target effects in future development.

### 4.5. Coronavirus Exoribonuclease as a Common Target for Antivirals

Unlike most RNA viruses, coronaviruses have the ability to edit mistakes during copying of their genetic information. The exoribonuclease domain of Nsp14 interacts with its cofactor Nsp10 to correct viral RNA mispairing by removing misincorporated nucleotides or nucleotide analogues from the 3′-end of the nascent RNA strand, a proofreading mechanism that is essential for the maintenance of the replicative fidelity of the coronavirus genome and is a candidate for antiviral drug targets for antiviral drugs [66]. Based on molecular docking technology, a number of Nsp14 inhibitors targeting the active site of exoribonuclease have been identified [67], such as nitrocatechol [68], SGC0946, and SGC8158 [69]. However, there is an off-target effect due to the structural similarity between Nsp14 and human DEDD exonuclease. Furthermore, in the absence of selective pressure, mutational inactivation of ExoN activity results in a 15-fold increase in mutation frequency across the coronavirus genome, posing an additional challenge for antiviral drug screening, which must be taken into account in drug design and mechanism of action studies.

## 5. Common Drug Targets against Coronaviruses Based on Viral Assembly and Release

**Figure 4:**
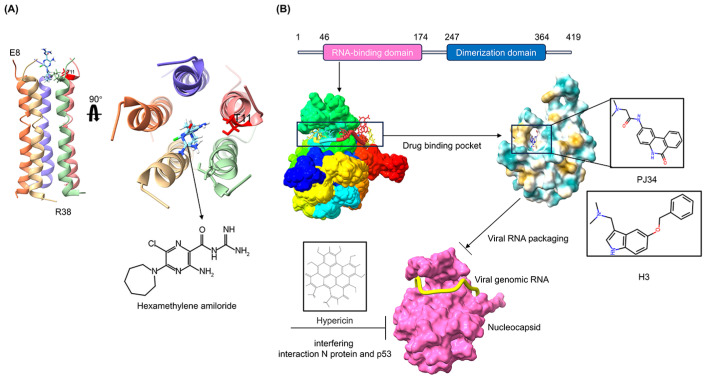
Common antivirals targeting structural proteins. (**A**) Pentameric oligomerization of transmembrane region (E8-R38) of the SARS-CoV-2 E protein (PDB ID 7K3G), and the co-crystal structure of hexamethylene amiloride with coronavirus envelope protein. (**B**) Structures of nucleocapsid and its drug-binding pockets (PDB ID 7ACT). The drug-binding pocket of the inhibitor PJ34 (PDB ID 4KXJ) or H3 is located within the interaction interface between the RNA-binding domain and viral single-strand RNA.

Antiviral drugs targeting the assembly as well as release of coronaviruses are mainly drugs designed and screened for coronavirus E and N proteins. Coronavirus E protein is a short polypeptide that increases membrane permeability to ions. This protein plays a key role in viral morphogenesis and assembly by interacting with other structural proteins [70]. E proteins can self-assemble into oligomers and generate ion channels that regulate the ionic homeostasis of the host cell and microenvironments. Blocking E protein channels can significantly reduce the pathogenicity of viruses [71]. In addition, E protein is also highly conserved in the viral subtypes, implicating E protein as a potential antiviral target. Hexamethylene amiloride (HMA) and amantadine (AMT), which target E protein pentameric ion channels, are broad-spectrum E protein inhibitors. Recent studies have shown that HMA exchanges ions between the helical chains of the SARS-CoV-2 E protein pentamer to close the N-terminal entrance of the protein, thereby blocking ion channel conductance (Figure 4A) [37]. AMT and its derivatives could inhibit the M2 ion channel activity of the influenza virus and have been used in the treatment of influenza A. Based on computer-aided drug design techniques, a variety of SARS-CoV-2 E protein inhibitors have been screened. These include retinoic acid, rutin, and nimbolin A [72]. Although these inhibitors have not been confirmed in vitro or in vivo, they have saved a great deal of money and time in the screening and identification of new drugs.

Coronavirus N protein is the most abundant protein in coronaviruses. The variety of functions include viral genome packaging and regulation of cell signaling pathways [38,73]. Coronaviruses can induce cell cycle arrest; during virion assembly, the PEDV N protein binds to p53 and then activates the p53-DREAM pathway, causing cell cycle arrest in the S phase. This arrest enhances viral replication. Some small-molecule compounds targeting coronavirus N proteins have recently been identified as candidate inhibitors of common antivirals (Figure 4B). For example, PJ34 and H3 inhibit the replication of HCoV-OC43 by interacting with S51, F53, R107, Y109, Y111, and R149 of N protein N-terminal domain (N-NTD) [74]. Notably, these amino acids are also conserved in SARS-CoV-2, suggesting a potential common antiviral possibility for both compounds. In addition, hyperoside, which targets the tertiary structure of the N^S171–N194^ domain, inhibits the replication of PEDV by antagonizing N protein-induced S phase arrest by interfering with the interaction between N protein and p53 [38]. These findings suggest that blocking the pathway by which coronaviruses regulate cell cycle arrest may be a strategy for the development of common antivirals. However, the development of N protein inhibitors remains a challenge. The drug-binding pocket in the N protein is structurally dynamic, which makes it difficult for drugs to bind stably, and the N protein is present in greater amounts in coronaviruses, leading to poor antiviral efficacy [75]. Despite active research being undertaken by investigators, no N protein inhibitors have been approved for use to date.

## 6. Conclusions and Prospects

Infectious diseases are among the major public health challenges. Outbreaks of viral infections in humans and animals have prompted a great deal of research on the pathogens of diseases worldwide [76]. There have been some encouraging advances in co-opted antiviral agents and a variety of approaches for designing anti-coronavirus strategies based on the mechanism of viral infection have been reported. However, many infectious diseases remain untreated [77]. In addition, emerging viral outbreaks pose a continuing threat to human health and life. Efficient and broad-spectrum inhibitors against viruses are powerful assets enabling rapid responses in the early stages of outbreaks and for preventative measures in the first instances of infection [78]. Therefore, the development of commensal antiviral drugs based on viral commensal mechanisms is of particular importance for dealing with emerging and outbreaks of infectious diseases [79].

Common antiviral drugs usually need to target critical proteins belonging to different viruses. Thus, in developing common antivirals, the basic biology of viruses must first be addressed to find suitable targets [80]. Based on the key aspects of the whole life cycle of infection, replication, and release of different RNA viruses, antiviral drug research has identified multiple common targets. These include S proteins targeting the entry into host cells, proteases targeting viral biosynthesis, and viral structural proteins targeting viral assembly and release, which can simultaneously inhibit the same or different coronaviruses. Thus, the combination of co-targeted drugs may enhance the effectiveness of antiviral drugs against each coronavirus type. In the future, if, after sequence identification, a new-onset virus is found to be from a known genus of viruses with a high degree of homology to existing viruses of the same genus, it may be treated by a stockpile of cocktail therapies targeting viruses of that genus [81]. The sequences and structures of proteins performing the same function are often highly similar across all viruses or in the same genus, and most antiviral drugs that are designed to target a conserved viral target have good antiviral efficacy against that virus as well as potential inhibitory effects on other viruses in the same genus or family [82]. Therefore, broad-spectrum viral inhibitors with conserved targets could be the first to be tried in cases of new outbreaks of viruses from known viral genera but with low homology to their homologs. In addition, based on the discovery of these new drug targets against the viral life cycle and their regulatory mechanisms, new drug targets with common antiviral effects and their precise structures and regulatory mechanisms can also be explored in future studies based on the characteristic molecular events in the replication process of other viruses.

Coronaviruses require the cooperative action of many host factors and cellular metabolic pathways to successfully infect host cells and effectively reproduce. Therefore, some critical host factors, such as ACE2, CD147, furin, cathepsin L, TMPRSS2, HSP90, HS, DC-SIGN/L-SIGN, SA, and TfR1, have also shown potential as antiviral targets [83,84,85]. For example, ACE2, CD147, and other functional receptors of coronaviruses can mediate the entry of viruses into host cells by binding to S proteins. Therefore, some antiviral drugs targeting functional receptors such as ACE2 can inhibit coronavirus replication in vitro by interfering with the binding of the virus to the receptor and affecting the invasion of the virus, thus exerting broad-spectrum antiviral effects. Similar to this mechanism of action, antiviral targets such as Furin, TMPRSS2, and other host cytokine proteases that mediate viral invasion can block the protein hydrolysis cleavage sites between the S1/S2 subunits of the viral S-protein, which are required for viral cell entry. Blocking the proteases necessary for these cleavages can affect the viral life cycle and pathogenicity, thereby inhibiting viral replication. Drugs targeting host cells can effectively inhibit the rapid replication of viral nucleic acids and combat viral drug-resistant mutations. However, broad-spectrum antiviral drugs targeting the host may be harmful to the host, and the in vitro inhibitory activity and in vivo therapeutic efficacy may not be fully consistent. In response to the host factors during viral infection and proliferation, the common principles, targeting, and safety of their inhibition of viral replication can be explored in the future to find new targets that are critical for viral replication but not for host cells. In this way, broad-spectrum and highly effective multi-targeted chemotherapy modalities can be designed to support chemical interventions.

The development of coronaviral antiviral drugs for multiple targets and multiple antiviral combinations, as well as broad-spectrum anti-coronaviral drugs for a common target, is a future research direction. Clarifying the mechanism of action of coronaviral antiviral drugs will further enhance and improve the drug design strategy for coronaviral antiviral drugs, facilitate screening for more efficient antiviral drugs, further enrich the potential of antiviral drug family to combat the current coronavirus pandemic and prevent future outbreaks of coronaviruses and the potential spillover of zoonotic coronaviruses, and provide basic support for the research and development of original RNA viral infectious disease. The collective knowledge also provides basic support for the development of original therapeutic drugs for RNA viral infectious diseases.

## Figures and Tables

**Table 1:** Antiviral drugs and antiviral targets.

Drugs	Class	Virus	Targets	EC_50_ (µM)	IC_50_ (µM)	Experimental Setting	Reference
FD001	Small-molecule compounds	SARS-CoV-2	S	1.58	0.18	In vitro	[24]
Arbidol	Indolyl carboxylic acid	SARS-CoV-2	S	4.11	/	In vitro	[25]
Phycobilin	Natural products	SARS-CoV-2	PLpro	/	62	In vitro	[26]
Adriamycin	Anthracycline	MERS-CoV	PLpro	/	1.67	In vitro	[27]
F0213	Lead inhibitor	SARS-CoV-2	PLpro	4.5	7.4	In vitro	[28]
Xanthohumol	Chalcones	SARS-CoV-2	3CLpro	5.93	1.53	In vitro	[29]
Hypericin	Anthraquinones	PEDV	3CLpro	3.53	5.09	In vitro	[30]
Tomatidine	Alkaloids	PEDV	3CLpro	/	3.45	In vitro	[31]
Octyl gallate	Aliphatics	PEDV	3CLpro	/	22.15	In vitro	[32]
Paxlovid	Compound antiviral drug	SARS-CoV-2	3CLpro	/	/	/	[33]
Remdesivir	Carboxylic ester	SARS-CoV-2	RdRp	0.62	0.65	In vitro	[34]
Gossypol	Gossypol	SARS-CoV-2	RdRp	0.31	0.76	In vitro	[35]
SSYA10-001	Heterocyclic compounds	SARS-CoV	RNA helicase	7	8.95	In vitro	[36]
Hexamethylene amiloride	Pyrazines	HCoV-229E	E	1.34	/	In vitro	[37]
Hypericin	Anthraquinones	PEDV	N	/	/	/	[38]

## Data Availability

Not applicable.
